# Suppression of Tumor Growth by *Pleurotus ferulae* Ethanol Extract through Induction of Cell Apoptosis, and Inhibition of Cell Proliferation and Migration

**DOI:** 10.1371/journal.pone.0102673

**Published:** 2014-07-16

**Authors:** Weilan Wang, Kaixu Chen, Qing Liu, Nathan Johnston, Zhenghai Ma, Fuchun Zhang, Xiufen Zheng

**Affiliations:** 1 Life Science and Technology, Xinjiang University, Xinjiang Key Laboratory of Biological Resources and Genetic Engineering, Urumqi, China; 2 Norman Bethune College of Medicine, Jilin University, Changchun, China; 3 Department of Pathology, Western University, London, Canada; 4 Lawson Health Research Institute, London, Canada; University of Toronto, Canada

## Abstract

Cancer is the second leading cause of death worldwide. Edible medicinal mushrooms have been used in traditional medicine as regimes for cancer patients. Recently anti-cancer bioactive components from some mushrooms have been isolated and their anti-cancer effects have been tested. *Pleurotus ferulae*, a typical edible medicinal mushroom in Xinjiang China, has also been used to treat cancer patients in folk medicine. However, little studies have been reported on the anti-cancer components of *Pleurotus ferulae*. This study aims to extract bioactive components from *Pleurotus ferulae* and to investigate the anti-cancer effects of the extracts. We used ethanol to extract anti-cancer bioactive components enriched with terpenoids from *Pleurotus ferulae*. We tested the anti-tumour effects of ethanol extracts on the melanoma cell line B16F10, the human gastric cancer cell line BGC 823 and the immortalized human gastric epithelial mucosa cell line GES-1 *in vitro* and a murine melanoma model *in vivo*. Cell toxicity and cell proliferation were measured by MTT assays. Cell cycle progression, apoptosis, caspase 3 activity, mitochondrial membrane potential (MMP), migration and gene expression were studied *in vitro*. PFEC suppressed tumor cell growth, inhibited cell proliferation, arrested cells at G0/G1 phases and was not toxic to non-cancer cells. PFEC also induced cell apoptosis and necrosis, increased caspase 3 activity, reduced the MMP, prevented cell invasion and changed the expression of genes associated with apoptosis and the cell cycle. PFEC delayed tumor formation and reduced tumor growth *in vivo*. In conclusion, ethanol extracted components from *Pleurotus ferulae* exert anti-cancer effects through direct suppression of tumor cell growth and invasion, demonstrating its therapeutic potential in cancer treatment.

## Introduction

Edible mushrooms have been globally used for centuries to promote health, prevent and treat diseases primarily via their vast medicinal qualities. There are more than 14,000 mushrooms, among which approximately 700 exhibit medicinal properties [1]. Medicinal mushrooms can improve cardiovascular health, stimulate host immune defense systems against viral and microbial infection and cancer, maintain glucose homeostasis and modulate detoxification [1]. They were used to treat many diseases such as atherosclerosis, hyperlipidemia, diabetes, hepatitis, and cancer [1]. The anti-cancer effects of mushroom species, or their constituent bioactive agents, have been tested against several major forms of human cancer in numerous experimental models including: stomach, breast, colon, lung, liver and skin. Researches on anti-tumor properties have primarily been focused on a small number of mushroom species such as *Ganoderma lucidum* (also known as Reishi in Japan or Lingzhi in China), and *Lentinus edodes* (Shiitake mushrooms) [2].

*Pleurotus ferulae* (PF) is an edible mushroom of the arid steppe and belongs to the family pleurotaceae and order agaricales [3]. As an aparasitic fungus, this edible mushroom grows on the living rhizome trunks of *Ferula asafoetida* in the Gobi desert and is mainly distributed in Xinjiang, China. PF produces various biologically functional components such as β-glucan, peptides, polysaccharides, organic acids, triterpenoids, mevinoli, saponins and steroids [4], [5], [6]. The mushroom has been traditionally used as a folk medicine for treating cancers. Recent studies have shown that PF exerts anti-oxidant [5], anti-hyperlipidemic [5], anti-tumor [6], immunomodulating [7], [8], anti-inflammatory and anti-microbial activities, as well as homeostasis of blood glucose [9]. The anti-tumor effects have been demonstrated in several human cancer cell lines such as the gastric cancer cell line MGC-803, cervical cancer cell line HeLa, and lung cancer cell lines A549 and SPC-a-1 *in vitro* [6]. On the other hand, the mushroom has not been shown to be toxic to normal liver cells [6]. Despite the clinical benefits and the therapeutic potential of *P. ferulae*, there have been only limited studies reported on its physiologically beneficial components.

Malignant melanoma is the most aggressive type of skin cancer with high metastatic potential and resistance to most treatments. Although melanoma makes up less than 5% of all skin cancers, it is responsible for a large majority of skin cancer fatalities [10]. Despite current advances in the development of new drugs and biochemotherapies, currently there is no effective treatment against invasive melanoma [11]. Therefore, a search for new therapeutic strategies with greater effectiveness and fewer side effects is necessary. We hypothesized that bioactive components from *P. ferulae* can suppress melanoma growth *in vitro* and *in vivo*.

In the present study, we extract bioactive components from *P. ferulae* using an ethanol extraction method and investigate its anti-tumor effect on the melanoma cell line B16F10 *in vitro* and a mouse melanoma model *in vivo*.

## Materials and Methods

### Materials and reagents

Fruiting body of *Pleurotus ferulae Lanzi* was purchased from Xinjiang, China. RPMI 1640 medium, Dulbecco’s modified Eagle medium and dimethyl sulfoxide (DMSO) were purchased from Gibco (Life Technology, Grand Island, NY). 3-(4, 5-Dimethylthyiazol-2-yl)-2,5-diphenyltetrazolium bromide (MTT) was purchased from Sigma (St. Louis, MO, USA). Penicillin/streptomycin was purchased from Invitrogen (Life Technology, Grand Island, NY). All the plates used in this study were purchased from Costar (Costar, USA).

### Animals

C57BL/6 female mice at the age of 6 weeks were purchased from the First Teaching Hospital of Xinjiang Medical University (Urimuqi, Xinjiang, China). All mice were maintained in the standard animal facility of Xinjiang University with a regular commercial diet. The experimental protocol was approved by the Animal Care and Use Committee of Xinjiang University.

### Extraction of bioactive component from *P. ferulae* using ethanol

100 g of fresh fruiting bodies of *P. ferulae* were purchased from China, cleaned with wet tissue paper without washing and sterilized by cleaning with an ethanol pad. Cleaned mushroom was sliced into 5 mm×10 mm chips and ground to a fine powder. The powder of PF fruit bodies was macerated three times with 1000 ml of 95% (v/v) ethanol with stirring at 50°C for 3 h, followed by a 30 minute sonication under 300 W at 25°C. The extracts were pooled together and were centrifuged at 3000 rpm for 15 min and then filtered through Whatman No. 4 filter paper. Ethanol was subsequently removed from the extract using a rotary vacuum evaporator at 40°C, and the remaining solvent was removed with a freeze-drier. Extracts used for *in vitro* assays were constituted in plain RPMI 1640 medium and sterilized with a 0.22 µm filter. The constituted extracts were further diluted with plain RPMI 1640 medium to certain concentrations just prior to use. Extracts used for *in vivo* assays were further diluted in PSB prior to use.

### Cell culture

The murine melanoma cell line B16F10, the human gastric cancer cell line BGC-823, cervical cancer Hela cells, breast cancer MCF-7 cells and the immortalized human gastric epithelial mucosa cell line GES-1 were purchased from the China Center for Type Culture Collection (CCTCC, Wuhan, China). Cells were cultured in RPMI 1640 medium containing 10% heat-inactivated fetal bovine serum (FBS) (Life Technology, Grand Island, NY), 100 U/ml of penicillin, and 100 µg/ml streptomycin at 37°C in a humidified incubator with 5% carbon dioxide (CO_2_) [13].

### *In vitro* cell toxicity and proliferation assay

The toxic effect of ethanol extracts from PF on cell proliferation of B16F10, BGC-823 and GES-1 were measured using a Dimethyl thiazolyl tetrazolium bromide (MTT) assay (Sigma, USA). MTT assay was performed by using the method of Akiyama *et al* [12] with slight modifications. Briefly, 4×10^3^ cells per well were cultured in a 96-well culture plate with RPMI 1640 medium, containing 10% FBS and antibiotics, overnight to reach 80% confluency. The cultures were washed with fresh RPMI 1640 once, treated with PFEC dissolved in RPMI 1640 medium at a variety of concentrations (0, 0.4, 0.8, and 1.6 mg/ml), and cultured at 37°C in 5% CO**_2_** and 95% air. Cisplatin was used as a positive control. 24, 48 or 72 hours after treatment, 200 µl of 1 mg/ml MTT solution was added to each well, and cultures were further incubated for 4 h before 200 µl of DMSO was added to the cultures to dissolve formed crystals. A micro plate reader was used to measure absorbance at 490 nm. Growth inhibition rate was calculated as follows:
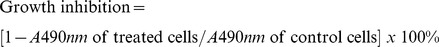



### Cell cycle analysis

Cellular DNA content and cell cycle distribution were ascertained by flow cytometric analysis after 24 h incubation of B16F10 cells with *P. ferulae* extracts [13]. Briefly, 4×10^5^ cells from control (untreated) and treated cells were harvested, washed twice with cold PBS, and fixed in 70% ethanol overnight. Next, fixed cells were incubated with propidium iodide (PI, 30 µg/ml) and RNase (20 µg/ml) for DNA staining and RNA degradation, respectively. After 30 min incubation, samples were subjected to flow cytometry analysis.

### Migration assay

The migration assay was conducted following the method of Lee *et al* [14] with some modifications. In brief, B16F10 cells (10^5^/well) were plated in a 6-well plate. After reaching 90% confluency, the center of the culture dishes was scratched once with a 200 µl pipette tip. Cells were washed three times and incubated with *P. ferulae* mushroom extract at concentrations of 0.8, and 1.6 mg/ml. 24 h after treatment, images of each well were taken under a microscope. The degree of cell spreading and directional migration was then compared. The relative migration rate was estimated by measuring the distance cells migrate for 24 h, as control cells cultured in full medium alone as a normalizer.

### Cell apoptosis by flow cytometry

B16F10 cells were harvested 24 h after treatment with 0.8, and 1.6 mg/ml *P. ferulae* extracts and stained with Annexin V-FITC and PI using a Chromatin Condensation/Dead Cell Apoptosis Kit (Invitrogen) according to the manufacturer’s instruction. Cell apoptosis was measured by flow cytometry.

### Mitochondrial membrane potential (MMP) assay

Mitochondrial transmembrane potential (ΔΨm) was measured to detect the damage of the mitochondria using flow cytometry for according to a previous study [15]. Cells were cultured and treated with PFEC for 48 h. Cells were then incubated with 30 nmol/L 3, 3-dihexyloxacarbocyanine iodide (DiOC6) (Molecular Probes, Eugene, OR, USA) for 15 min in dark conditions at 37°C. Changes in fluorescent DiOC6 intensity were analyzed by flow cytometry.

### Caspase 3 activity assay

Colorimetric caspase 3 assay kit (Bebio, Shanghia, China) was used for determination of caspase 3 activity in B16F10 cells after treatment with *P. ferulae* extracts. This assay is dependent on the hydrolysis of the peptide substrate acetyl-Asp-Glu-Val-Asp-p-nitroaniline (Ac-DEVD-pNA) by caspase 3, resulting in the release of the p-nitroaniline. Caspase 3 activity was measured following the manufacturer’s instruction [12]. Briefly, the cells were treated and incubated for 24 h at 37°C in a 5% CO_2_ atmosphere. Cells were harvested and cell pellets were lysed in 1 x lysis buffer on ice for 10 min. The lysates of cells were centrifuged at 12,000 rpm for 10 min. 5 µg of the supernatant was mixed with 85 µl of assay buffer and 10 µl of caspase 3 substrate and reacted at 37°C for 2 h. For each sample, the concentrations of the pNA released from the substrate were calculated from their absorbance values at 405 nm using an ELISA reader.

### *In vivo* mice experiment

The *in vivo* anti-tumor effect of *P. ferulae* extract was investigated in a mouse melanoma model. C57BL/6 female mice at the age of 6 weeks were randomly divided into three groups. Each group consisted of ten mice. Group 1, as a negative control, was treated with PBS only; Group 2, P. ferulae ethanol extracted components (PFEC) treatment group, was orally treated with PFEC (1000 mg/kg per day) daily using an intragastric syringe from the day of tumor cell inoculation; Group 3 (Cisplatin treatment) was treated with intraperitoneal injection of Cisplatin (100 mg/kg per day) diluted in PBS. All mice were subcutaneously injected with B16F10 cells (2×10^5^ cells/mouse). The treatment started on the day of tumor cell inoculation and continued for 12 days. The body weight of mice was measured and tumor growth was monitored every 3 days until all mice were sacrificed. Animals were euthanized in a CO chamber 20 days after tumor cell injection. Tumor tissues were collected and weighed.

### Gene expression by reverse transcriptase -quantitative polymerase chain reaction (qRT-PCR)

Total RNA was extracted from cells using a RNeasy Mini Kit (Qiagen, Germany). cDNA was synthesized with 2 µg total RNA using oligo (dT) and reverse transcriptase (Invitrogen, Life Tech) as described by manufacturer’s instruction. Sequences of primers used in this study were as follows: AKT, 5′-CCACGCTACTTCCTCCT-3′ (forward) and 5′-CCGCTCTGTCTTCATC-3′ (reverse), PI3K, 5′-CCTGCTCCGTAGTGGTA-3′ (forward) and 5′-TTCATCGCCTCTGTTGTG-3′ (reverse); Bcl-2, 5′-CACTTGCCACTGTAGAGA-3′ (forward) and 5′-GCTTCACTGCCTCCTT-3′ (reverse); Bcl-xL, 5′-GTGGCTGGTGTGGTT-3′ (forward) and 5′-GTAGTGGTTCTCCTGGTAG-3′ (reverse); Bax, 5′-GCCTCCTCTCCTACTTC-3′ (forward) and 5′-CCTCAGCCCATCTTCTT-3′ (reverse); Bid, 5′-CAGTCACGCACCATCTT-3′ (forward) and 5′-GGCTCCTCAGTCCATCT-3′ (reverse); Cyclin D1, 5′-AGAAGTGCGAAGAGGAG-3′ (forward) and 5′-GGATAGAGTTGTCAGTGTAGAT-3′ (reverse); Proliferating cell nuclear antigen (PCNA), 5′-TTGCACGTATATGCCGAGAC-3′(forward), and 5′- GGTGAACAGGCTCATTCATCTCT-3′ (reverse); and GAPDH, 5′-GGTGAAGCAGGCATCT-3′ (forward) and 5′-TGTTGAAGTCGCAGGAG-3′ (reverse).

Gene expression was detected by quantitative PCR using a SYBR green master mix kit (Qiagen, Germany) according to the manufacturer’s protocol. A primer concentration of 100 nmol/L was used in the PCR reaction. PCR reaction was conducted in an Applied Biosystems 7500 PCR instrument (Applied Biosystems, Carlsbad, CA) using the following conditions: 95°C for 30 sec, 59°C for 30 sec and 72°C for 30 sec, 40 cycles.

### Western blotting

40 µg of total proteins extracted from cells were loaded and separated on 12% PAGE and then transferred to PVDF membranes. The transferred membranes were blocked with 1x TBS containing 5% milk for 30 min at room temperature and blotted with the first antibodies against AKT, Bad, caspase 8, caspase 9 or GAPDH overnight at 4°C, respectively. All antibodies were purchased from Signaling transduction technology (Danvers, MA) and diluted in 1∶2000. The blotted membranes were washed with 1 X TBS-T (0.5% Tween-20) three times, followed by blotting with the HRP-conjugated secondary antibody for 30 min at room temperature. The membranes were developed by ECL and images of protein bands were taken using an ImageQuant LAS 4000mini system (GE Healthcare Life Sciences, Pittsburgh, PA).

### Statistical analysis

All data are presented as the mean ± S.E.M and experiments were repeated at least three times. Statistical analysis of the results was conducted using one-way ANOVA. The results were considered significant at a value of *p<0.05, **p<0.01 and ***p<0.001 versus the vehicle-treated control group.

## Results

### *P. ferulae* ethanol extracted components (PFEC) suppresses tumor cell growth and inhibits cell proliferation

**Figure 1:**
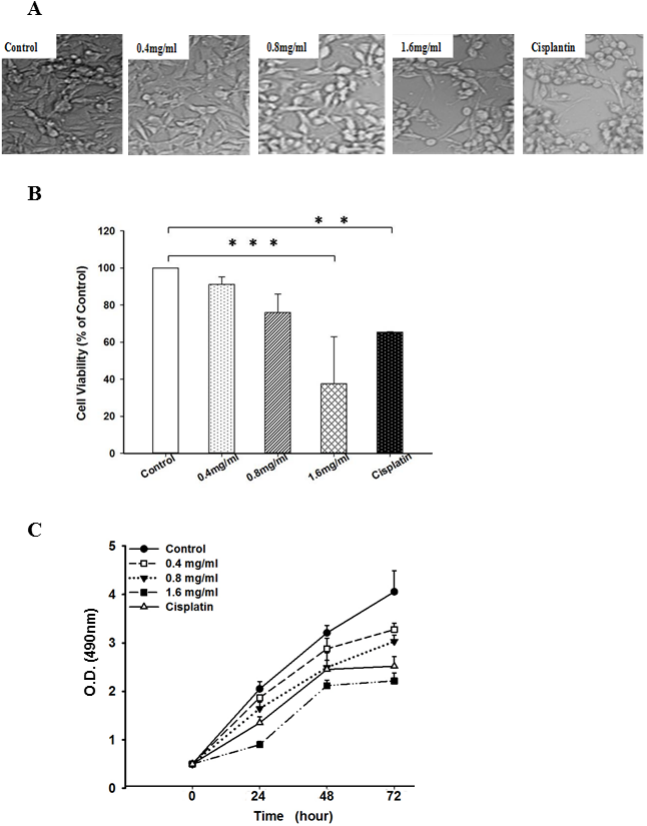
Toxicity of PFEC on B16F10 cells *in vitro*. *(* ***A*** *)* Cell morphology changes in B16F10 cells. B16F10 cells were cultured and treated with PF ethanol extracts at various concentrations or Cisplatin (20 µg/ml) for 24 h. Cells grown in complete medium only were used as control. Cell morphological change was imaged under phase contrast microscopy (×200). *(* ***B*** *)* Cell viability. Cells were treated as in (A). 24 h after treatment, cell viability was detected by MTT assay. The relative cell viability was calculated using control (culture with complete medium only) as a normalizer. **p<0.005, ***p<0.001. *(* ***C*** *)* Cell proliferation curve. Cell proliferation at 24, 48 and 72 hours after treatment (A), measured by MTT assays.

It is documented that bioactive components including terpenoids, which possess anti-cancer function, can be extracted from edible mushrooms by ethanol fraction extraction [16], [17]. Accordingly, we used 95% ethanol to extract bioactive components from PF. The yield of the PF extract was 6% (w/w). To investigate anti-tumor effects of PFEC, we first examined its effects on cell growth of the melanoma cell line B16F10 cells *in vitro*. Cultured B16F10 melanoma cells were treated with various concentrations of PFEC. 24 h later, cell morphologic changes were examined under a phase contrast microscope. As shown in Figure 1A, cells cultured with complete medium (control) displayed very nice and normal spindle shape and reached 90% confluency 24 h after culture. Cells treated with 0.4 mg/ml of PFEC looked similar to the control cells having nice spindle shape except with less cell numbers. However, cells treated with 0.8 mg/ml of PFEC started to shrink and lose their spindle shape and the cell confluency was reduced. More round, shrunken cells were observed in cells treated with 1.6 mg/ml of PFEC. In the culture with 3.2 mg/ml of PFEC, more cell debris was observed and the cell confluency was significantly lower (data not shown). Cells treated with Cisplatin changed into a round shape and were evidently dying. We also used MTT assays to detect viable cells. The number of dead cells increased as the concentration of PF extracts increased (Figure 1B). The concentration of PFEC that resulted in a 50% reduction in absorbance (IC50) compared with the control B16F10 cells after 24 h of treatment was 1.4 mg/ml.

Uncontrolled cell proliferation is a hallmark of cancer cells. We next investigated whether PFEC affected B16F10 cell proliferation. As shown in the cell proliferation curves (Figure 1C), control cells treated with complete RPMI 1640 proliferated over the observed time periods. In contrast, cell proliferation was reduced in cells treated with PFEC at all tested concentrations, as well as in cells treated with Cisplatin (a chemodrug). Cells treated with higher concentrations of PFEC proliferated less. The reduction in cell proliferation was observed starting from 24 h after treatment and became more pronounced as treatment time was extended. Taking RPMI 1640 control cells without PFEC or Cisplatin treatment as 100% growth and proliferation, we also calculated inhibition rates. Treatment with 0.4 mg/ml of PFEC showed 8.57%, 13.11% and 23.54% inhibition rate whereas 12.57%, 32.06% and 38.51% were seen for 0.8 mg/ml of PFEC at 24, 48 and 72 hours, respectively. PFEC inhibited 45% ∼60% of cell proliferation at the concentration of 1.6 mg/ml. The inhibitory effect of PF extract on cell proliferation was dose and time dependent.

**Figure 2:**
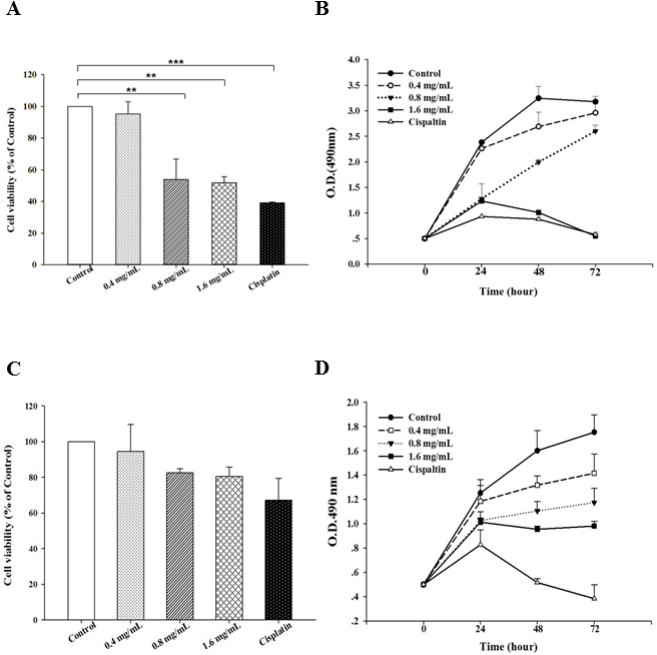
Toxicity of PFEC on human gastric cancer cell BGC-823 and human immortal gastric cells GES-1. *(* ***A*** *)* Cell viability of BGC-823. Cells were treated as in Figure 1. 24 h after treatment, cell viability was detected by MTT assay. The relative cell viability was calculated using control (culture with complete medium only) as a normalizer. *(* ***B*** *)* Cell proliferation. BGC-823 cells were cultured and treated with PFEC or Cisplatin. Cell proliferation at 24, 48 and 72 hours was detected as described in Figure 1. *(* ***C*** *)* Cell viability of GES-1 cells. Immortalized human gastric epithelial mucosa GES-1 cells were cultured and treated with PFEC, cell viability was measured by MTT assays. *(* ***D*** *)* Cell proliferation curve of GES-1 cells. GES-1 cells were cultured and treated with PFEC, cell viability was detected using MTT assays. Data shown is a summary of three independent experiments. **p<0.005 and ***p<0.001.

In order to explore the broadness of its anti-tumor effect, we treated human gastric cancer BGC-823 cells, cervical cancer Hela cells, breast cancer MCF-7 cells and immortalized human gastric GES-1 cells with PFEC and detected cell viability and cell proliferations. As shown in Figure 2, the cell viability was significantly lower in the human gastric cancer BGC-823 cells treated with 0.8 mg/ml and 1.6 mg/ml PFEC as compared with the RPIM 1604 control group (Figure 2A). Cell proliferation was also decreased in the BGC 823 cells treated with PFEC (Figure 2B). In contrast, cell viability and cell proliferation of GES-1 were not significantly decreased (Figure 2C and 2D), indicating that toxicity of PFEC is specific to cancerous cells. Moreover, the toxicity of PFEC was also evidenced on other cancer cell lines such as the human cervical cancer Hela cells and the human breast cancer MCF-7 cells (data not shown).

### PFEC arrests cells at G0/G1 phases

**Figure 3:**
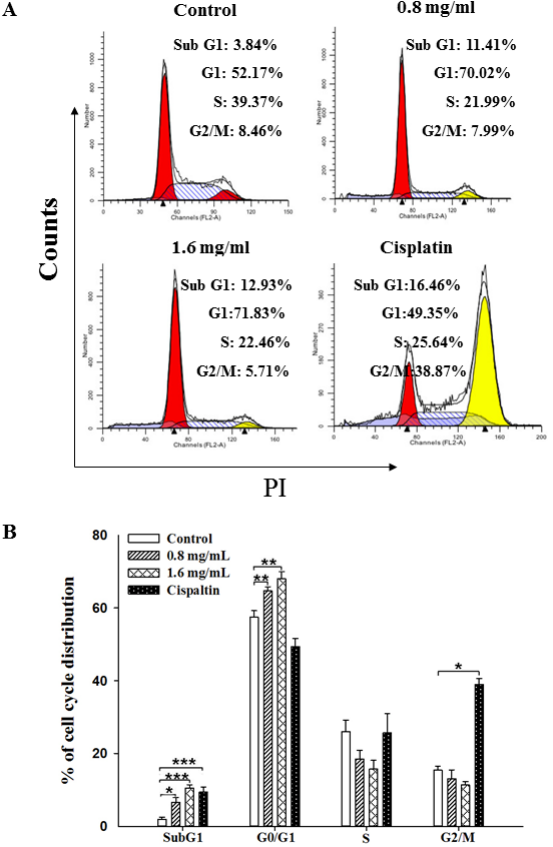
The effects of PFEC on the cell cycle of B16F10 cells. The cells were treated with various doses of PFEC or Cisplatin for 24*(* ***A*** *)*. The distribution of cell cycle stages in B16F10 cells was summarized in *(* ***B*** *)*. Each value was represented as the mean ± SEM of three independent experiments. *p<0.05, **p<0.005, ***p<0.001.

The induction of cell cycle arrest has been considered a major cause of anti-proliferation [18]. Since PF extracts exerted an anti-proliferative effect, we accordingly determined cell cycle distribution by flow cytometry to further elucidate the effect of PFEC on B16F10 cell cycle progression. Representative histograms for cell cycle distribution in B16F10 cells exposed to different concentrations of PFEC were shown in Figure 3A. The effects of PFEC on cell cycle distribution are also summarized in Figure 3B. Treatment of B16F10 cells with PFEC (1.6 mg/ml) for 24 h resulted in an increase of G0/G1 phase (71.4%) as compared to the media control (62.9%). This increase was accompanied with the decreased percentage of cells in S phase. After treatment for 24 h, the percentage of 0.8 mg/ml of PFEC-treated cells in S phase was 14.2%, whereas it was 19.2% in the control cells. In addition, flow cytometry analysis revealed the effect of PFEC on the induction of apoptotic cell death; an increase of cell percentage in the sub-G0/G1 phase. As shown in Figure 3A and 3B, the number of cells arrested at G0/G1 fraction were increased in PFEC-treated cells and the increase was dose-dependent. The positive control drug Cisplatin also significantly increased the sub-G0/G1 fraction (Figure 3A and B). These data suggest that the inhibition of cell proliferation in B16F10 cells by *P. ferula* extract may be exerted by the induction of G0/G1 phase arrest.

### PFEC induces cell apoptosis and death and increases caspase 3 activity

**Figure 4:**
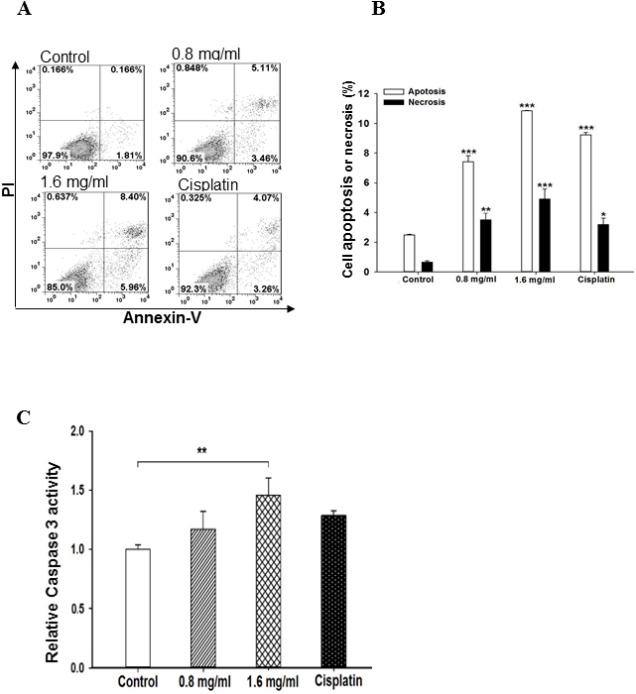
PFEC induced cell apoptosis and increase caspase 3 activity. B16F10 cells were double stained with Annexin V-FITC and PI and analyzed by flow cytometry following exposure to PFEC (0.4, 0.8, and 1.6 mg/ml) for 24 h. Cisplatin (20 µg/ml) was used as a positive control for apoptosis of B16F10 cells. *(* ***A*** *)* Representative flow cytometry data of cell apoptosis. *(* ***B*** *)* Summarized data of cell apoptosis. Each value was presented as mean ± SEM of three independent experiments. *(* ***C*** *)* Caspase 3 activity was increased by PF ethanol extracts. B16F10 cells were cultured and treated with PF ethanol extracts at the indicated concentrations. 24 h after treatment, cells were harvested for caspase 3 activity measurement. *p<0.05, **p<0.005, ***p<0.001.

Next, we investigated whether PFEC induced tumor cell apoptosis and death. To do so, we treated B16F10 cells with various concentrations of PFEC for 24 h. We detected cell apoptosis and death by double staining cells with Annexin V and PI. The intensity of fluorescence was measured by flow cytometry. As shown in Figure 4A and 4B, treatment with 0.8, and 1.6 mg/ml PFEC increased cell apoptosis/death. The higher the concentration of PFEC used the more apoptotic cells were observed.

During apoptosis, several caspase proteins are converted from the inactive (pro-form) to the active form. Caspase 3 is the major executioner caspase in the caspase apoptotic cascade. Therefore, we determined whether the induction of apoptosis by PFEC was exerted through the activation of caspase 3. As shown in Figure 4C, caspase 3 activity was increased to 1.2 fold by 0.8 mg/ml of PFEC, 1.5 fold by 1.6 mg/ml of PFEC and 1.28 fold by Cisplatin as compared with the control media treatment.

### PFEC reduces the mitochondrial membrane potential (MMP) of B16F10 cells

**Figure 5:**
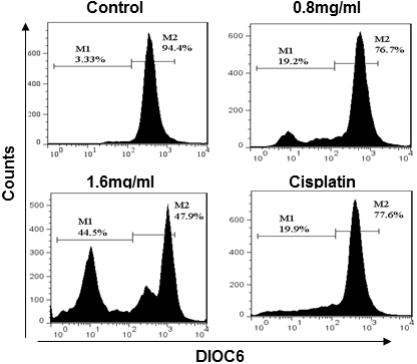
PF ethanol extracts reduced the mitochondrial membrane potential of B16F10 cells. B16F10 cells were treated with DiOC6 and the intensity of DiOC6 was measured by flow cytometry. Data were representative of three independent experiments.

The mitochondria is a major target of most anti-cancer drugs since damage of the mitochondria results in cell apoptosis and cell death. In order to understand whether PFEC induced cell apoptosis through disruption of mitochondrial membrane integrity, we detected MMP of B16F10 cell after treatment with PFEC by measuring changes of DiOC_6_ positive cells. DiOC6 is a positively charged lipophilic fluorochrome and can accumulate inside intact mitochondria. However, cells with damaged or leaky mitochondrial membranes cannot accumulate DiOC6 and thus they have low DiOC 6 fluorescence. As shown in Figure 5, 94% of normal cells without any PFEC treatment were DiOC6 positive, while only 76.7% and 47.9% DiOC6 positive cells were observed in the cells treated with 0.8 and 1.6 mg/ml PFEC, respectively. Cisplatin treatment reduced DiOC6 positive cells to 77.6%. The higher concentration of PFEC was applied onto the cells, the greater reduction in DiOC6 fluorescence intensity was observed, indicating more damage was induced on the mitochondria.

### PFEC prevents B16F10 cell invasion *in vitro*

**Figure 6:**
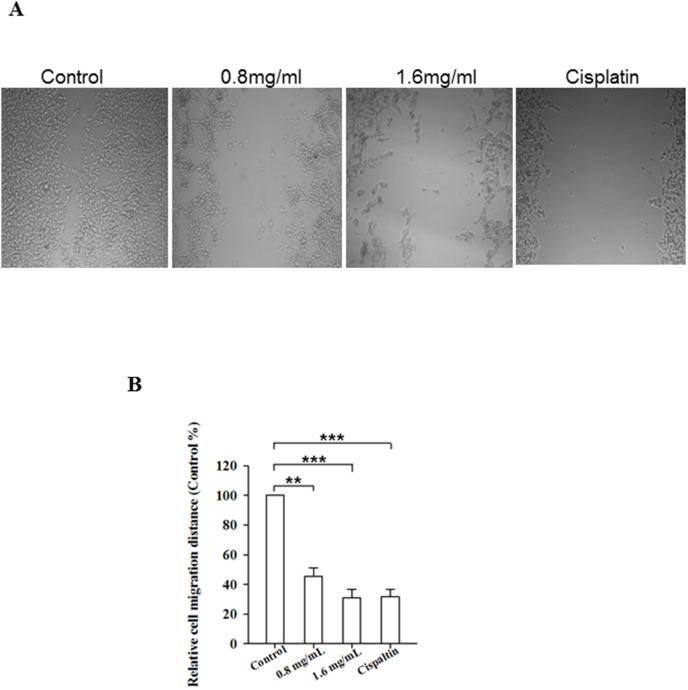
PF ethanol extracts inhibited tumor cell migration *in vitro*. B16F10 cells were plated and cultured overnight to reach ∼80% confluency. Cells were scratched with a 200 µl of pipette tip and then washed with culture medium. Fresh culture media containing a variety of PFEC were added to the cells. Cells were allowed to grow for an additional 24 h. The cell migration distances were imaged under a phase contrast microscope (x 40). *(* ***A*** *)* Representative images of cell migration. *(* ***B*** *)* Relative cell migration rate. The distance of cell migration overnight was measured. The relative migration rate was calculated as compared with the control group. Data is a summary of three independent of experiments. *p<0.05.

Cell invasion is another hallmark of tumor cells. To test the effect of PFEC on cell migration, we performed a scratch assay *in vitro*. Cells were cultured overnight to reach 80% confluency. Cells in the plate were scratched by a 200 µl pipette tip in the center of the well and treated with various concentrations of PFEC. The migration of cells was imaged as shown in Figure 6A and 6B. Cells in the control group (complete medium) filled the scratched space after the overnight culture, while treatment with PFEC reduced cell migration evidenced by the wider open space left between two sides of the cells. The inhibitory effect of PFEC on cell migration was dose-dependent. The combinational treatment with Cisplatin and PFEC reached the highest inhibitory effect on cell migration (Data not shown).

### PFEC delays tumor formation and reduces tumor growth *in vivo*

**Figure 7:**
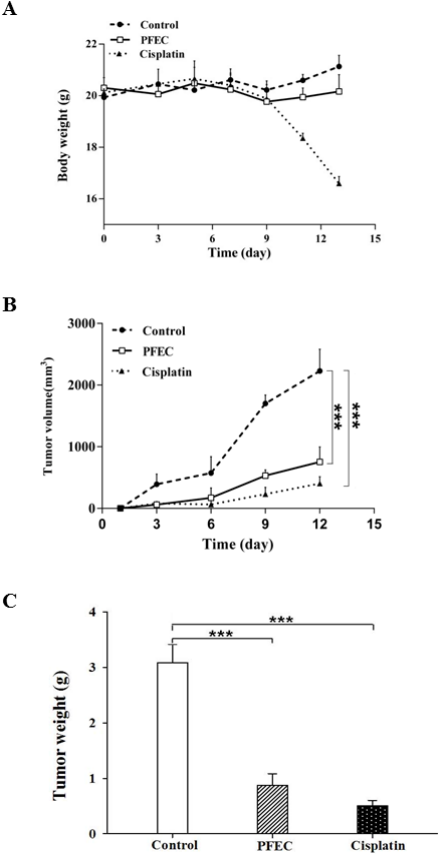
PF suppressed melanoma growth *in vivo*. *(* ***A*** *)* Body weight of mice. C57BL/6 mice (n = 10/group) were inoculated with B16F10 melanoma cells. On the same day, mice were treated with PBS, PFEC, or Cisplatin. Body weight of mice was measured every three days using a scale. *(* ***B*** *)* Tumor growth curve. Tumor size was measured every three days with a caliper. *(* ***C*** *)* Tumor weight. At the end point of the experiments, tumor tissues were collected and weighed. ***p<0.001.

Given PFEC prevents tumor cell proliferation, inhibits cell migration and enhances cell apoptosis, we explored the potential of PFEC in preventing and treating tumors *in vivo*. We treated mice with PFEC daily by oral administration for 12 days after tumor cell inoculation. All mice looked healthy without any abnormal behaviors throughout the entire treatment period. The bodyweight of mice did not significantly change over time between PBS control groups and PFEC treatment group (Figure 7A), despite that the Cisplatin treated group started to lose body weight on day 10 after treatment. Histopathological analysis showed that there were no pathological changes in the liver organs (data not shown), indicating there is no obvious toxicity of PFEC to animals. In contrast, tumors in both PFEC treated mice and Cisplatin treated mice grew slower than the control PBS treated group (Figure 7B). At the endpoint of experiments, mice treated with PFEC had smaller tumors as compared with the PBS control group (Figure 7C). Overall, daily oral administration of PFEC after B16F10 inoculation reduced tumor growth, tumor size and final tumor weight.

### PFEC changes the expression of genes associated with apoptosis and the cell cycle

**Figure 8:**
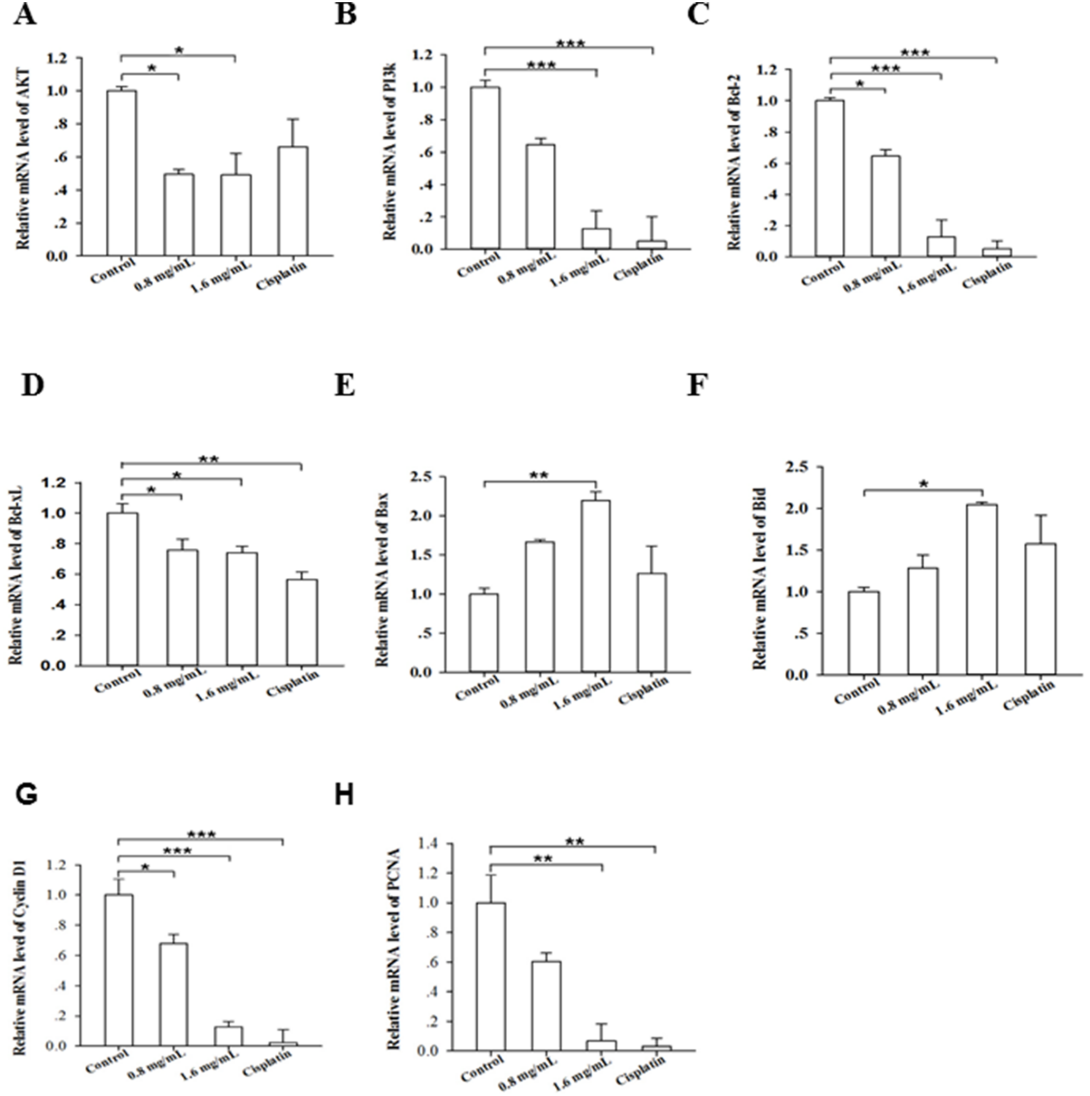
PFEC regulated gene expression *in vitro.* B16F10 cells were plated and treated with PFEC. 24-PCR. Gene expression was calculated using ΔΔCt and normalized with the RPMI 1640 treated group. GAPDH was used as an internal control. *(* ***A*** *)* Akt; *(* ***B*** *)* PI3K; *(* ***C*** *)* Bcl-2; *(* ***D*** *)* Bcl-xL; *(* ***E*** *)* Bax; *(* ***F*** *)* Bid, (*G*) Cyclin D1, and (*H*) PCNA. Experiments were repeated three times independently. *p<0.05, **p<0.005, ***p<0.001.

In order to understand how PFEC induced apoptosis of tumor cells and inhibited cell proliferation, we detected gene expression of cell growth and anti- and pro-apoptosis genes such as Akt, PI3K, Bcl-2, Bcl-xL, Bax, Bid and cell proliferation gene cyclin D1 and PCNA in cells by quantitative PCR. We found that treatment with PFEC decreased the expression of gene Akt (Figure 8A), PI3K (Figure 8B), Bcl-2 (Figure 8C) and Bcl-xL (Figure 8D), while increased the expression of pro-apoptotic genes BAX (Figure 8E) and Bid (Figure 8F) in B16F10 cells *in vitro*. We also observed that PFEC treatment reduced the expression of cyclin D1 (Figure 8G) and PCNA (Figure 8H) in cells.

**Figure 9:**
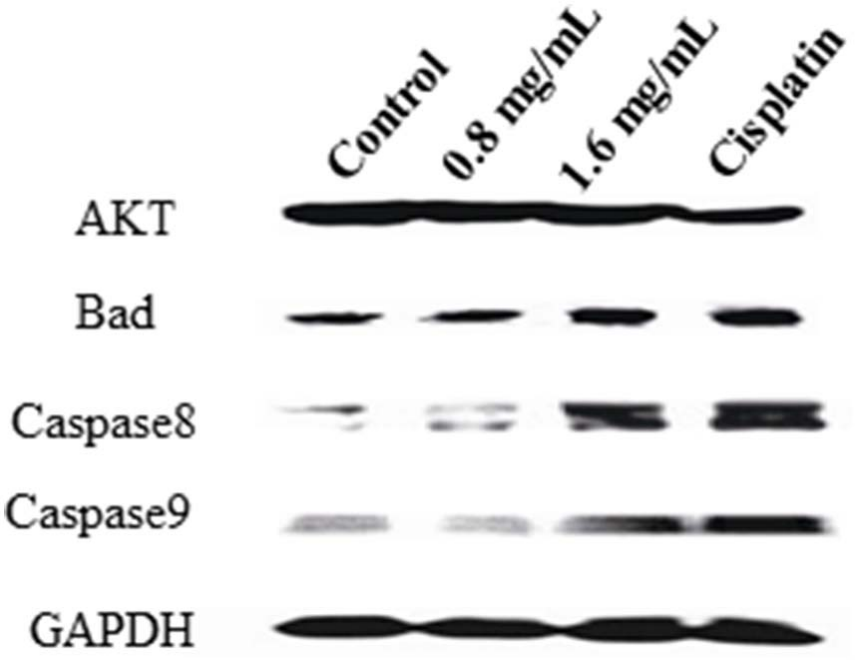
PFEC increased Bad, caspase8 and caspase 9 expression in cells at the protein level, but decreased AKT expression. B16F10 cells were treated with PFEC as described in Figure 8. 24 h after treatment, total proteins were extracted from the treated cells and subjected to Western blotting for detecting the expression of Akt, Bad, caspase 8 and caspase 9. Data present one of three repeated experiments.

Additionally, we also detected the expression of AKT, Bad, caspase 8 and caspase 9 at the protein level. As shown in Figure 9, PFEC treatment decreased AKT expression, while increased the expression of apoptosis related gene Bad, caspase 8 and caspase 9. Taken together, these results indicated that PFEC inhibits tumor growth through regulating the expression of genes associated with cell apoptosis, proliferation and growth.

## Discussion

In this study, we demonstrated for the first time that: 1) PFEC inhibited B16F10 melanoma tumor cell growth by induction of cell apoptosis and inhibition of cell proliferation *in vitro*; 2) PFEC reduced tumor cell migration *in vitro*; 3) PFEC reduced tumor growth *in vivo*, showing its promise as a treatment for melanoma; and 4) PFEC had no significant toxicity in the animals.

Edible mushrooms enrich nutrients and bioactive components and have been used to treat diseases such as diabetes, cancer and cardiovascular disease for ages in Asia [1], [2]. Nevertheless, an entire mushroom undergoing no extraction process was used for tradition medicine [19]. In advance of biotechnologies, it is possible to isolate and purify bioactive components to augment the therapeutic effects of the mushroom and to understand its functional mechanisms [16], [17]. The extraction and isolation of bioactive constituents from mushrooms for therapies is still young. In general, there are two common methods used to extract raw bioactive components–hot water and ethanol or methanol extraction. Hot water extraction is for extracting water soluble materials such as polysaccharides, oligosaccharides, protein and enzymes [20], [21], [22], [23]. In contrast, ethanol or methanol is used for extracting terpenoids, alkaloids, etc [16], [17], [24]. In this study, we used a three time ethanol extraction to obtain crude products. The yield of products increased as the percentage of ethanol used increased. The highest yield of products was 6% (W/W) achieved using 95% ethanol. Sonication increased the release of components from the mushroom. We thus used the products from 95% ethanol extraction with sonication in this study. Although the active components in PFEC that exert the anti-tumor effects are not yet identified, the product is abundant in terpenoids, but not protein nor saccharides determined by a series of qualitative chemical reaction assays (data not shown), which is in line with other types of mushroom [25].

Terpenoids are a superfamily of naturally existing small organic chemicals [26]. Plant terpenoids are used widely in traditional herbal remedies and their anti-bacterial, anti-cancerous and other pharmaceutical functions are under investigation [27]. It has been demonstrated that terpenoids extracted from mushrooms possess anti-tumor effects by directly suppressing cancer cell growth and invasion [28]. Among terpenoids, triterpenoids are ones that possess anti-tumor effects, which are mediated through inhibition of tumor cell proliferation and induction of apoptosis. In this study, we observed that treatment with PF extract comprising a high percentage of terpenoids inhibited B16F10 cell growth in a dose and time dependent manner. The effects of PFEC were competitive to the currently used chemotherapeutic drug Cisplatin, and the effects were even better than Cisplatin when observed at the high concentrations of 1.6 and 3.2 mg/ml. The suppression of cell growth was seen in other human cancer cells such as human gastric cancer BGC 823 cells and breast cancer cells (data not shown), implying a broad application of the PF extract for treating cancers. In addition to inhibiting cell growth, we also found that PF ethanol extract reduced B16F10 cell migration *in vitro*, which is in agreement with results from other mushrooms [29]. In contrast, PFEC did not significantly affect the growth of normal non-malignant cells, such as normal gastric cells, and was not toxic to those normal cells. Our *in vivo* studies have also shown that PFEC did not affect body weight of animals and did not shown any obvious side effects.

We found that the suppression of cancer cell growth was associated with the inhibitory effect of PF extract on cell proliferation as well as the induction of cell apoptosis. PF extracts also arrested B16F10 cell proliferation in the G0/G1 phase of the cell cycle and reduced the portion of cells in the mitotic/dividing phases (S and G2/M phase), which agrees with previous studies from other mushroom extracts [30]. The expression of cell cycle genes such as cyclin D1 and PCNA were reduced by PF extracts, which further confirmed the effects of PF extract on cell cycle arrest and proliferation.

Cell apoptosis is one of the events occurring in cancer cells after being exposed to chemodrugs [5]. There are two types of death pathways in apoptosis: the extrinsic pathway and the intrinsic mitochondria-mediated pathway. The intrinsic pathway is triggered by multiple stimuli, which causes mitochondrial membrane permeabilization, leading to the release of pro-apoptotic mitochondrial proteins from the mitochondria into the cytosol. Consequently, this activates caspase-dependent and caspase-independent pathways and induces cell apoptosis and necrosis. In this study, we observed that PFEC increased apoptotic cells *in vitro*. Moreover, PFEC reduced the mitochondrial membrane potential and increased caspase 3 activities. These results suggest that PFEC induced apoptosis is mediated through the intrinsic pathway, which is similar to most chemodrugs. The gene expression data showed that PFEC induced cell apoptosis by down-regulating the expression of anti-apoptotic genes Bcl-2 and Bcl-xL and cell growth gene Akt, PI3k, and up-regulating pro-apoptotic genes Bax, Bid, Bad, caspase 8 and caspase 9.

*In vivo* studies showed that the ethanol extract of PF slowed down tumor growth and reduced tumor burden, indicating its anti-tumor effect *in vivo*. Meanwhile, we observed that the bodyweight did not significantly change between groups over time and the animals did not display abnormal behaviors or signs, thus implying low or no toxicity towards animals or normal tissue. Our data is consistent with previous reports that show low or no toxicity to normal liver cells [30]. Nevertheless, a further study on its toxicity is needed to better address safety issues of the extract for future clinic usage.

Additionally, in traditional medicine most mushrooms are taken by people after multiple soakings in hot water. Major components are water soluble, among which proteins and polysaccharides exert anti-tumor effects [33], [34]. In our studies, we also used the hot water method to extract bioactive components from PF. We found that water extracts of PF contained proteins and polysaccharides and suppressed a variety of human and murine cancer cell growth (data not shown). Taken together, PF, like other well-studied medicinal mushrooms, is comprised of terpenoids as well as polysaccharides and proteins, and is a good resource for the development of natural anti-cancer agents.

In conclusion, ethanol extracts of PF exert an anti-cancer effect through direct induction of cell apoptosis and death, inhibition of cell proliferation and reduction in cell migration. Our results demonstrated the potential of PF extract as an agent for treating cancer patients.
